# Proteomics analyses of acute kidney injury biomarkers in a rat exertional heat stroke model

**DOI:** 10.3389/fphys.2023.1176998

**Published:** 2023-06-12

**Authors:** Fu-Li Wen, Yong-Jun Xu, Lai-En Xue, Yun-Feng Fu, Lin-Lin Cui, Jun-Zhu Wang, He-Ping Zheng, Dong-Hui Zhou, Jun Lu

**Affiliations:** ^1^ Fujian Provincial Key Laboratory of Transplant Biology, 900 Hospital of the Joint Logistics Team, Fuzong Clinical Medical College of Fujian Medical University, Fuzhou, China; ^2^ Center for Experimental Research in Clinical Medicine, Fujian Provincial Hospital, Shengli Clinical Medical College of Fujian Medical University, Fuzhou, China; ^3^ Key Laboratory of Fujian-Taiwan Animal Pathogen Biology, College of Animal Sciences (College of Bee Science), Fujian Agriculture and Forestry University, Fuzhou, China

**Keywords:** exertional heatstroke, acute kidney injury, proteomics, Acsm2, AHSG, biomarkers

## Abstract

The frequency of exertional heat stroke (EHS) increases with the gradual elevation of global temperatures during summer. Acute kidney injury (AKI) is a common complication of EHS, and its occurrence often indicates the worsening of a patient’s condition or a poor prognosis. In this study, a rat model of AKI caused by EHS was established, and the reliability of the model was evaluated by HE staining and biochemical assays. The expression of kidney tissue proteins in the EHS rats was analyzed using label-free liquid chromatography–tandem mass spectrometry. A total of 3,129 differentially expressed proteins (DEPs) were obtained, and 10 key proteins were finally identified, which included three upregulated proteins (Ahsg, Bpgm, and Litaf) and seven downregulated proteins (medium-chain acyl-CoA synthetase 2 (Acsm2), Hadha, Keg1, Sh3glb1, Eif3d, Ambp, and Ddah2). The qPCR technique was used to validate these 10 potential biomarkers in rat kidney and urine. In addition, Acsm2 and Ahsg were double-validated by Western blotting. Overall, this study identified 10 reliable biomarkers that may provide potential targets for the treatment of AKI caused by EHS.

## 1 Introduction

Heat stroke is a life-threatening illness caused by an imbalance between heat production and heat loss in the body due to exposure to high temperatures and/or strenuous exercise. It is characterized by an increase in core temperature (Tc) exceeding 40°C and central nervous system abnormalities, such as altered mental status, convulsions, coma, and multi-organ damage (Leon and Bouchama, 2015; Bouchama et al., 2022). In accordance with different causes and different susceptible populations, heat stroke is divided into classic heat stroke and exertional heat stroke (EHS) (Bouchama and Knochel, 2002). EHS has become one of the most common diseases among military personnel, workers, and athletes (Mao et al., 2016). The Armed Forces Health Surveillance Bureau reported 1,560 cases of heat stroke and 6,055 cases of heat exhaustion among active-duty U.S. military personnel who served from 2018 to 2020 (bureau, 2019, 2020, 2021).

Most patients with EHS have kidney injuries, which is associated with a variety of factors, including direct heat injury, pre-kidney damage due to volume deficit, kidney perfusion deficit, rhabdomyolysis, and disseminated intravascular coagulation (Jung et al., 2020). A total of 25%–35% of patients with EHS would develop acute oliguric kidney failure (AUSTIN and BERRY, 1956). Acute kidney injury (AKI), one of the serious complications of EHS, has an incidence up to 50% and is life-threatening in patients with severe conditions; however, its specific molecular mechanisms have not been fully determined (AUSTIN and BERRY, 1956).

Serum creatinine is an important indicator for the assessment of AKI, and its expression level is influenced by several non-kidney factors, such as age, gender, muscle mass, diet, and nutritional status (Huang et al., 2017). The lack of early diagnostic markers is one of the major causes of delayed treatment initiation, especially in the treatment of AKI caused by EHS. Previous studies have shown that L-FABP can be used to monitor patients with EHS to identify individuals who need immediate treatment to prevent serious kidney damage (Goto et al., 2023). In recent years, proteomics technologies have been widely used to screen biomarkers for the diagnosis and treatment of various diseases, and research on kidney injury has rapidly developed (Arthur et al., 2018; Dubin and Rhee, 2020). Currently, no studies have been reported regarding the use of proteomics to treat AKI caused by EHS, and the biomarkers of this disease are yet to be discovered and explored.

**FIGURE 1:**
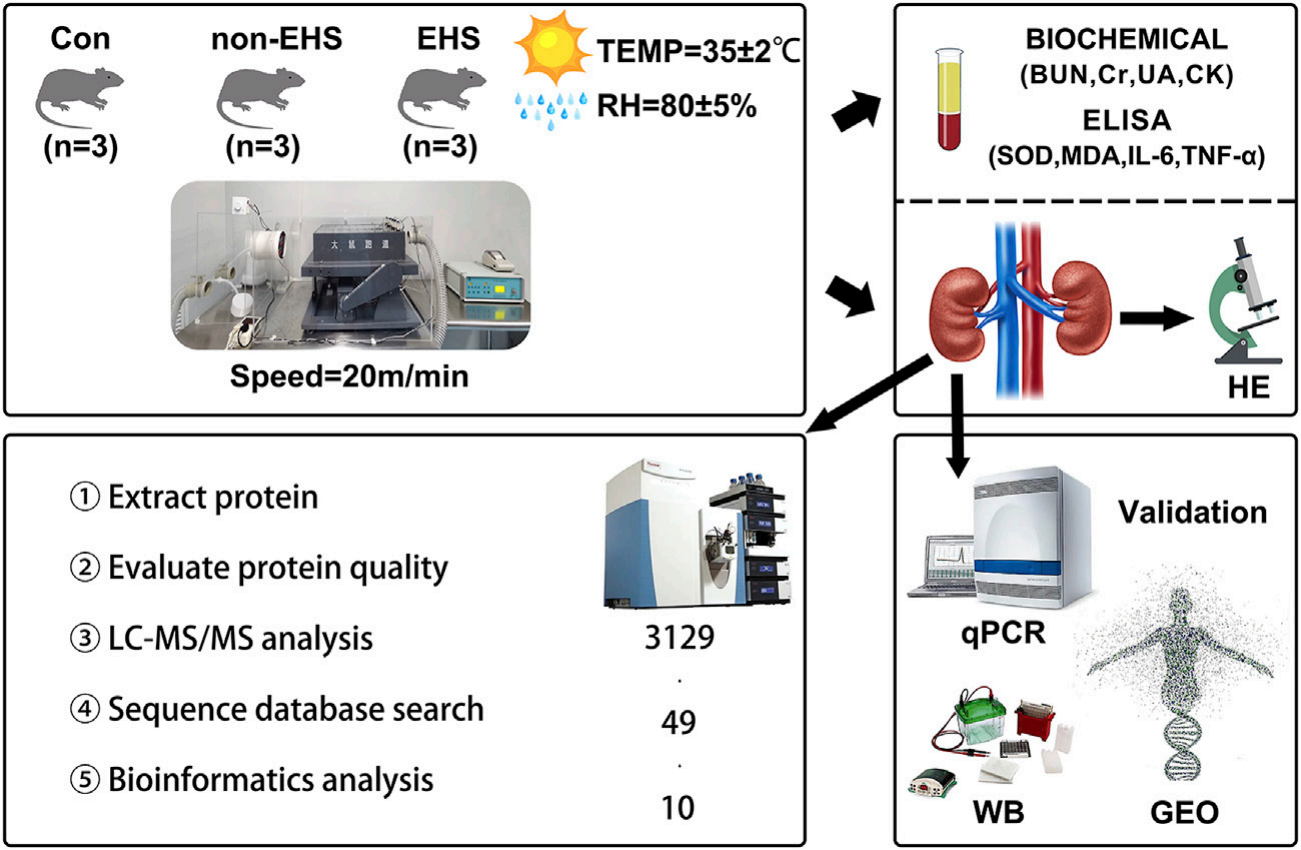
Overview of the study. Briefly, a rat model of AKI caused by EHS was established, and rat blood was collected for biochemical and ELISA assay analysis. Then, proteomic analysis of rat kidneys was performed, and 10 candidate biological targets that were identified using qPCR and WB were screened. Finally, additional validation was performed using the GEO database of human acute kidney injury.

In this study, a rat model of EHS was established, and label-free quantitative proteomics were utilized to identify the protein expression changes involved in the process of AKI caused by EHS. Ten candidate biomarkers were screened and identified to provide potential therapeutic targets for this disease, laying a foundation for further in-depth study of the underlying mechanism of this disease (Figure 1).

## 2 Materials and methods

### 2.1 Animals

All animal studies were approved by the Experimental Animal Welfare and Ethics Committee of the 900 Hospital of the Joint Logistics Team, PLA (Fuzhou, China). Thirty-six male Sprague–Dawley rats (age, 12 weeks; weight, ∼300 g) were provided by Shanghai SLAC Laboratory Animal Co., Ltd., Shanghai, China. The rats were housed in cages under controlled humidity (55% ± 5%), light (12 h/12 h light/dark cycle), and temperature (22°C ± 2°C) with free access to water and food.

### 2.2 Experimental design

After 7 days of adaptive feeding, the rats were randomly divided into three groups. Rats in the control group (Con group, n = 36) were untreated, and rats in the no-EHS group (non-EHS group, n = 36) and the EHS group (EHS group, n = 36) underwent 5 days of adaptive load-increasing training. On the first day, the rats were trained at a speed of 10 m/min for 10 min. Their running speed was increased by 2 m/min per day, and their running time was increased by 5 min per day. On day 5, the rats were trained at the speed of 20 m/min for 30 min. In addition, all of the trained rats were given 2 days to recover. On day 8, the rats in the non-EHS and EHS groups were tested at the constant speed of 20 m/min in an environment with normal temperature and relative humidity (TEMP = 22°C ± 2°C, RH = 55% ± 5%) and an environment with a high temperature and relative humidity (TEMP = 35°C ± 2°C, RH = 80% ± 5%). Tc was measured every 10 min, and the rats were given electrical shocks of 1.00 mA when they were not running. The rat model of EHS was considered to be successfully established when the Tcs of the rats reached 42.9°C ± 0.1°C. Three rats in each group were randomly selected to obtain kidneys for proteomic assays and the rest were used for the next experiments.

### 2.3 Sample preparation

All the rats were anesthetized with 1% sodium pentobarbital (40 mg/kg) via intraperitoneal injection. Heart blood was then collected and centrifuged at 1,200 *g* for 10 min at 4°C to separate the serum. The urine was withdrawn from the bladder using a syringe, centrifuged at 5000rpm for 5 min, the supernatant was carefully discarded, 0.1 mL of physiological saline was added and the mixture was shaken. All blood samples and urine samples were separated within 1 h, which was stored at −80°C. The rats were dissected to obtain kidneys. One kidney cortical tissue sample was stored in a refrigerator at −80°C and used for proteomics, RT-qPCR, and Western blot (WB) analysis. The other kidney sample was placed in 4% paraformaldehyde and utilized for HE staining.

### 2.4 Biochemical assays

Blood urea nitrogen (BUN), serum creatinine (Cr), uric acid (UA), and creatine kinase (CK) were measured with a fully automated biochemical analyzer (Chemray 800, China). The reagents for these assays were purchased from Shenzhen Radu Life Science Co.

### 2.5 HE staining

Paraformaldehyde-fixed kidney tissues were paraffin-embedded and cut into 4 µm slices. Dewaxing was performed with xylene and gradient ethanol (100%, 95%, 80%, and 70%) and was followed by staining with 10% hematoxylin and 1% eosin. The slices were further dehydrated and then sealed by using neutral gum. Finally, the slices were observed using a biological microscope (DM 2000; Leica Microsystems GmbH).

### 2.6 Nano-LC-MS/MS analysis

The kidney tissues of 3 rats in each group were randomly selected for proteomics. For each sample, 1 µg of total peptides were separated and analyzed with a nano UPLC (EASY- nLC1200) coupled to a Q Exactive HFX Orbitrap instrument (Thermo Fisher Scientific) with a nano-electrospray ion source. Separation was performed using a reversed phase column (100 μm ID ×15 cm, Reprosil Pur 120 C18 AQ, 1.9 μm, Dr. Maisch). Mobile phases were H_2_O with 0.1% FA, 2% ACN (phase A) and 80% ACN, 0.1% FA (phase B). Separation of sample was executed with a 120 min gradient at 300 nL/min flow rate. Gradient B: 2%–5% for 2 min, 5%–22% for 88 min, 22%–45% for 26 min, 45%–95% for 2 min, 95% for 2 min. Data dependent acquisition (DDA) was performed in profile and positive mode with Orbitrap analyzer at a resolution of 120,000 (at200 m/z) and m/z range of 350–1,600 for MS1; For MS2, the resolution was set to 15,000 with a dynamic first mass. The automatic gain control (AGC) target for MS1 was set to 3E6 with max IT 50 m, and 1E5 for MS2 with max IT 110 m. The top 20 most intense ions were fragmented by HCD with normalized collision energy (NCE) of 27%, and isolation window of 1.2 m/z. The dynamic exclusion time window was 45 s, single charged peaks and peaks with charge exceeding 6 were excluded from the DDA procedure. This study was performed by Biotree Biomedical Technology CO., LTD. (Shanghai, China).

### 2.7 Proteome discoverer database search

Vendor’s raw MS files were processed using Proteome Discoverer (PD) software (Version 2.4.0.305) and the built-in Sequest HT search engine. MS spectra lists were searched against their species-level UniProt FASTA databases (uniprot-Rattus + norvegicus-10116-2020-10. fasta), Carbamidomethyl [C] as a fixed modification, Oxidation M) and Acetyl (Protein N-term) as variable modifications. Trypsin was used as proteases. A maximum of 2 missed cleavage(s) was allowed. The false discovery rate (FDR) was set to 0.01 for both PSM and peptide levels. Peptide identification was performed with an initial precursor mass deviation of up to 10 ppm and a fragment mass deviation of 0.02Da. Unique peptide and Razor peptide were used for protein quantification and total peptide amount for normalization. All the other parameters were reserved as default.

### 2.8 Quantitative RT-PCR

RNA was extracted from kidney tissues and urine in accordance with the instructions of the RNA extraction kit (cat. no. ET111-01, Trans Gen and cat. no. R1300, Solarbio, respectively). cDNA was obtained in accordance with the instructions of the reverse transcription kit (cat. no. RR047A, Takara). cDNA was diluted 5-fold and β-actin (rat) was used as an internal reference gene to detect the relative expression of the gene. Nucleic acids were amplified using a qPCR instrument (TOWER3G, Analytik Jena). All primers were designed with Primer 5.0 (Table 1). Relative fold changes in mRNA expression were calculated with the Equation 2^−ΔΔ^CT.

**TABLE 1:** Primer sequences for the RT-qPCR of each gene.

Gene name	Primer sequence 5′–3′	Primer sequence 3′–5′	Product size (bp)
*Acsm2*	CCA​CTT​CAT​GGG​ACG​GAC​A	TTT​CAA​CCA​CGG​CAG​GAT​GT	104
*Hadha*	GAT​CCT​AGC​CAC​GCC​TGA​AG	GCT​GAC​ACG​GGG​TAA​ACT​GT	170
*Sh3glb1*	GTA​ATC​ACC​TGT​CCT​CCT​AAC​CT	TAG​CCA​GTC​GGA​GTC​CAT​TC	199
*Eif3d*	TTG​TCC​AGA​GGG​TCG​GAT​CT	TCT​CTC​CCC​ATT​CTC​AGG​CA	196
*Ahsg*	ACG​ATG​CCC​AAT​CCT​GAT​CC	GAC​CAG​TAC​AGT​CAG​TCG​CA	191
*Ambp*	TTC​GCC​TCC​GAG​AAG​GAA​TG	GCT​GCA​TCA​AAT​GCC​CAG​AG	113
*Litaf*	TCC​AGG​ACC​TTA​CCA​AGC​AG	GGG​CGT​TGG​GTA​GTA​ACT​GT	100
*Bpgm*	GCG​TCA​CTA​TGG​AGC​CTT​GA	CAC​GTC​GCA​CAC​TTT​GTA​CC	175
*Ddah2*	ACT​TCG​CTG​TCT​CTA​CGG​TAC	ATC​AGT​CAG​TGC​TGC​CAT​TG	140
*β-actin*	CGC​GAG​TAC​AAC​CTT​CTT​GC	CCT​TCT​GAC​CCA​TAC​CCA​CC	211

Note: Premier 5 used in primer design.

### 2.9 WB analysis

Frozen kidney tissue specimens were removed from liquid nitrogen and placed on ice for grinding. After washing with PBS, the supernatant was discarded through centrifugation, and 0.4 mL of RIPA lysate (10 µL of phosphatase inhibitor and 5 µL of protease inhibitor) was added to the precipitate. The suspension was placed on ice for 20 min and subjected to vortex shaking at 3 min intervals. The suspension was then centrifuged at 12,000 *g* and 4°C for 20 min. The resulting supernatant was used for protein quantification via the BCA method. It was separated via 10% SDS-PAGE, and then transferred to a PVDF membrane, which was then placed in a blocking solution containing 5% skim milk powder and blocked at room temperature for 2 h. The membrane of internal reference protein membrane (β-actin, 1:3000, cat. no. ab227168, Abcam) and the membrane of target protein membrane (Acsm2a, 1:1000, cat. no. 22862-AP, Proteintech; Ahsg, 1:2000, cat. no. PB0598, Boster) were placed in 1% BSA diluted solution and incubated overnight at 4°C. The secondary antibody was left at room temperature for 1 h (horseradish enzyme-labeled goat anti-rabbit IgG, 1:5000, cat. no. ab6721, Abcam). A mixture with equal volumes of liquid A and liquid B of the Thermo ECL kit (cat. no. 34080, Thermo) was added to the membrane surface. The membrane was then left in the dark for 3 min. Subsequently, the luminescent solution was removed from the membrane and placed on a transparent membrane. Luminescence was detected using a chemiluminescence system, and images of the bands were collected. ImageJ was utilized to analyze the grayscale values of the bands.

### 2.10 ELISA

Total superoxide dismutase (SOD), malondialdehyde (MDA), tumor necrosis factor α (TNF-α), and interleukin 6 (IL6) were detected using a multifunctional enzyme detector (Epoch, United States). Kits were purchased from the Nanjing Institute of Biological Engineering. All assays were performed in accordance with the instructions for the use of the kits.

### 2.11 Statistical analysis

Statistical analyses were performed using the SPSS software version 20.0 (SPSS, Inc., Chicago, IL, United States). Data are expressed as the mean ± SD, and were analyzed using one-way ANOVA followed by Bonferroni pairwise comparisons. *p* < 0.05 was considered a significant differenc.

## 3 Results

### 3.1 Effects of EHS on the kidneys of rats

**FIGURE 2:**
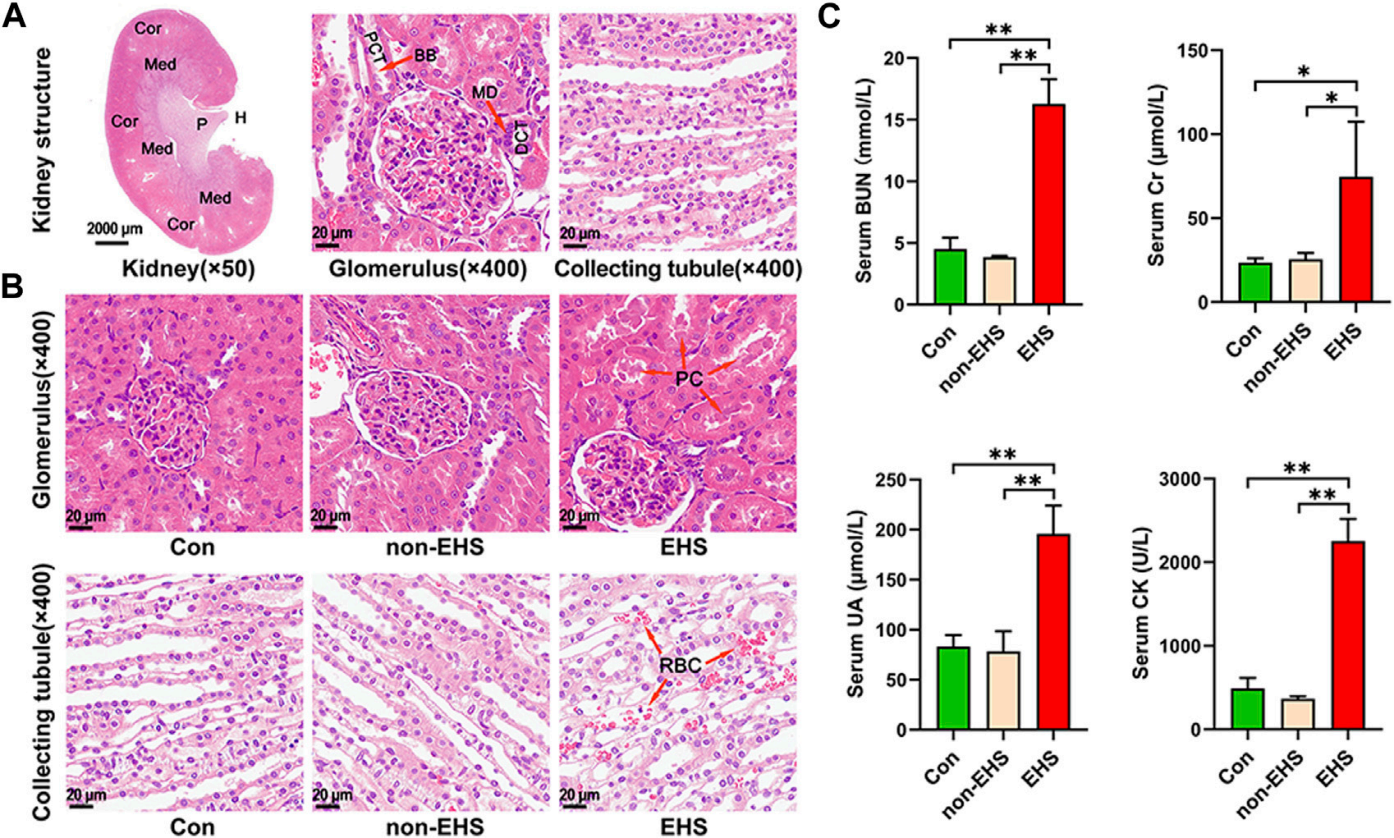
Effects of EHS on the kidney of rats. **(A)** HE staining of kidneys. **(B)** HE staining of glomeruli and collecting ducts. **(C)** Serum biochemical assay of rats, one-way ANOVA, *p < 0.05, **p < 0.01 (biological replicate = 12, technical replicate = 3).

The Tcs of all of the EHS group rats reached 42.9°C ± 0.1°C. Moreover, the rats experienced clinical symptoms, such as poor mental status, loss of appetite, disheveled fur, and slowed response to external stimuli. The HE-stained photographs of the entire kidney tissue were scanned, and the glomeruli and collecting tubules were visualized (Figure 2A). HE staining revealed extensive hemorrhages, swelling of tubular epithelial cells, vacuolar degeneration, and protein cast in the kidney tubules of the rats in the EHS group (Figure 2B). By contrast, no significant abnormalities were observed in the kidneys of the Con and non-EHS groups. The serum biochemical tests show that the level of BUN, Cr, UA, and CK were significantly increased in the EHS group but did not significantly changed in the Con and non-EHS groups (Figure 2C). These results suggest that the rat model of AKI caused by EHS was successfully established.

### 3.2 Acquisition of differentially expressed proteins in the kidneys of rats with EHS

**FIGURE 3:**
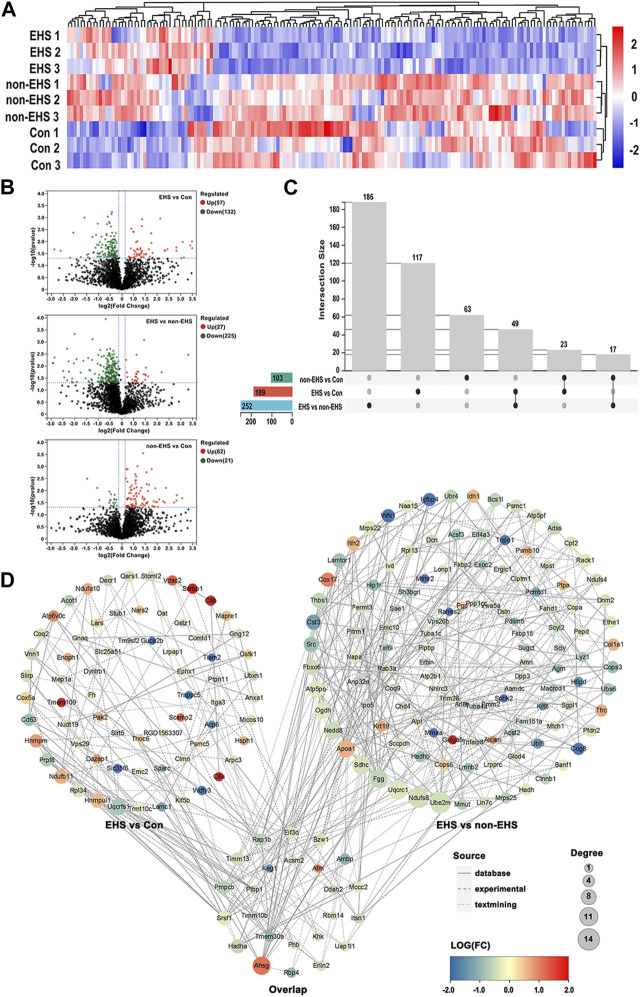
DEPs acquisition in the kidney of rats with EHS. **(A)** Heat map of DEPs. **(B)** Volcano plot of DEPs. **(C)** Venn diagram drawn by UpSetR. **(D)** PPI network plot. Overlap group contains the same DEPs in the PPI network between EHS vs. Con group and EHS vs. non-EHS group.

The differentially expressed proteins (DEPs) in kidney tissues from rats in the EHS, non-EHS, and Con group were analyzed using label-free liquid chromatography–tandem mass spectrometry, and a total of 3,129 DEPs were obtained. The DEPs were analyzed using hierarchical clustering and visualized in the form of a heat map (Figure 3A). Statistical processing with Student’s t-test was carried out to screen for DEPs with the criteria of *p* < 0.05 and fold changes of <0.83 or >1.2. The results of DEP screening were visualized in the form of a volcano plot (Figure 3B). A data comparison revealed that 49 identical DEPs were screened between the EHS vs. non-EHS group and EHS vs. Con group (Figure 3C). Then, further cross-validation with proteomic databases was performed. A protein–protein interaction (PPI) network was built using STRING 11.5. The protein interaction network graph was constructed using Cytoscape software (Figure 3D), and the results of betweenness, closeness, and degree for each protein were analyzed using the CytotoNCA app. Ten candidate DEPs were finally identified including three upregulated proteins (Ahsg, Bpgm, and Litaf) and seven downregulated proteins (Acsm2, Hadha, Keg1, Sh3glb1, Eif3d, Ambp, and Ddah2) (Table 2).

**TABLE 2:** Biomarkers for screening after proteomic analysis of kidney tissue in a rat model of AKI caused by EHS.

GeneName	Accession	Coverage [%]	# Peptides	# PSMs	# Protein unique peptides	# Unique peptides	# AAs	MW [kDa]	Calc. pI	Score	Description
Acsm2	O70490	43	18	92	0	17	572	64.1	8.31	139.94	Acyl-coenzyme A synthetase ACSM2, mitochondrial OS = *Rattus norvegicus* OX = 10,116 GN = Acsm2 PE = 2 SV = 2
Hadha	Q64428	35	22	96	20	22	763	82.6	9.06	95.57	Trifunctional enzyme subunit alpha, mitochondrial OS = *Rattus norvegicus* OX = 10,116 GN = Hadha PE = 1 SV = 2
Ahsg	P24090	26	8	44	1	8	352	38	6.51	58.58	Alpha-2-HS-glycoprotein OS = *Rattus norvegicus* OX = 10,116 GN = Ahsg PE = 1 SV = 2
Ambp	Q64240	15	5	27	5	5	349	38.8	6	51.44	Protein AMBP OS = *Rattus norvegicus* OX = 10,116 GN = Ambp PE = 1 SV = 1
Bpgm	Q7TP58	12	5	19	0	5	395	45.3	6.92	2.44	Phosphoglycerate mutase OS = *Rattus norvegicus* OX = 10,116 GN = Bpgm PE = 1 SV = 1
Eif3d	Q6AYK8	9	5	19	5	5	548	63.9	6.05	11.18	Eukaryotic translation initiation factor 3 subunit D OS = *Rattus norvegicus* OX = 10,116 GN = Eif3d PE = 1 SV = 1
Keg1	Q9Z2Y0	15	4	9	3	3	295	34	8.78	9.21	Glycine N-acyltransferase-like protein Keg1 OS = *Rattus norvegicus* OX = 10,116 GN = Keg1 PE = 1 SV = 2
Ddah2	Q6MG60	16	4	20	3	3	285	29.7	6.01	24.65	N(G),N(G)-dimethylarginine dimethylaminohydrolase 2 OS = *Rattus norvegicus* OX = 10,116 GN = Ddah2 PE = 1 SV = 1
Sh3glb1	Q6AYE2	2	1	8	1	1	365	40.8	6.04	15.49	Endophilin-B1 OS = *Rattus norvegicus* OX = 10,116 GN = Sh3glb1 PE = 2 SV = 1
Litaf	B2RYP2	4	1	4	1	1	161	17	5.88	0	LPS-induced TN factor OS = *Rattus norvegicus* OX = 10,116 GN = Litaf PE = 2 SV = 1

### 3.3 Functional analysis of DEPs in the kidneys of rats with EHS

**FIGURE 4:**
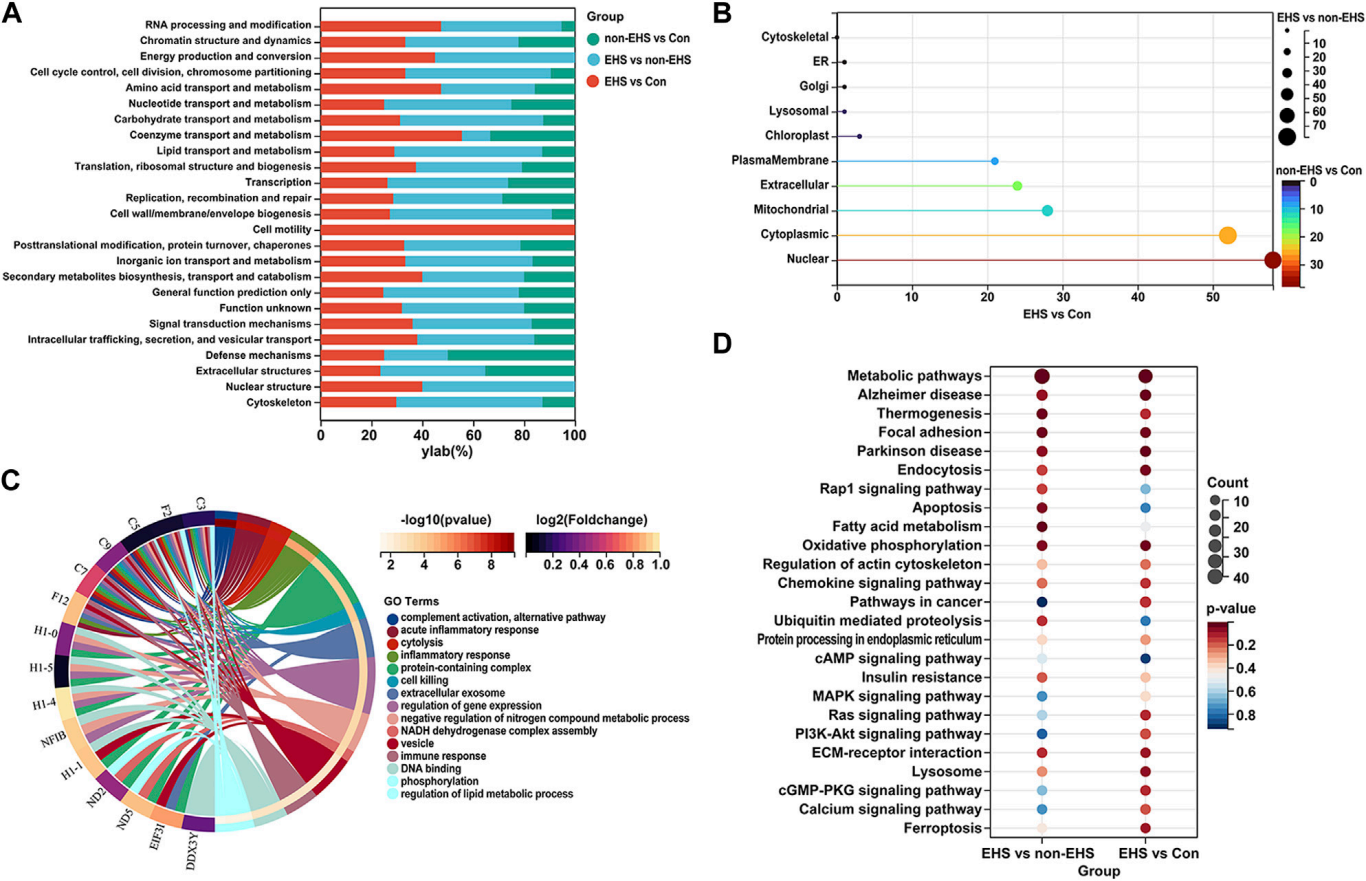
Functional analysis of DEPs in the kidney of rats with EHS. **(A)** COG (KOG) analysis of DEPs. **(B)** Subcellular localization of DEPs. **(C)** GO annotation enrichment analysis of DEPs. **(D)** KEGG annotation enrichment analysis of DEPs.

The COG (KOG) database was used to analyze the percentage of proteins in different functional categories that reflect the metabolic or physiological bias in the corresponding period and environment. The results show that the top five DEPs were involved in signal transduction, post-translational modification, protein turnover, chaperones, general function prediction, intracellular trafficking, secretion, vesicular transport, and the cytoskeleton (Figure 4A). Subcellular localization was used to accurately determine the possible sites where the DEPs executed their functions. The results show that the top five cellular locations wherein the DEPs functioned were cytoplasmic, nuclear, mitochondrial, extracellular, and plasma membrane locations (Figure 4B). The GO (Figure 4C) and KEGG (Figure 4D) annotation enrichment analysis of the DEPs was performed using Sangerbox tools. The results show that the DEPs play a major role in mitochondrial energy metabolism, inflammatory response, and apoptosis.

### 3.4 Validation of DEPs in the kidney and urine of rats with EHS

**FIGURE 5:**
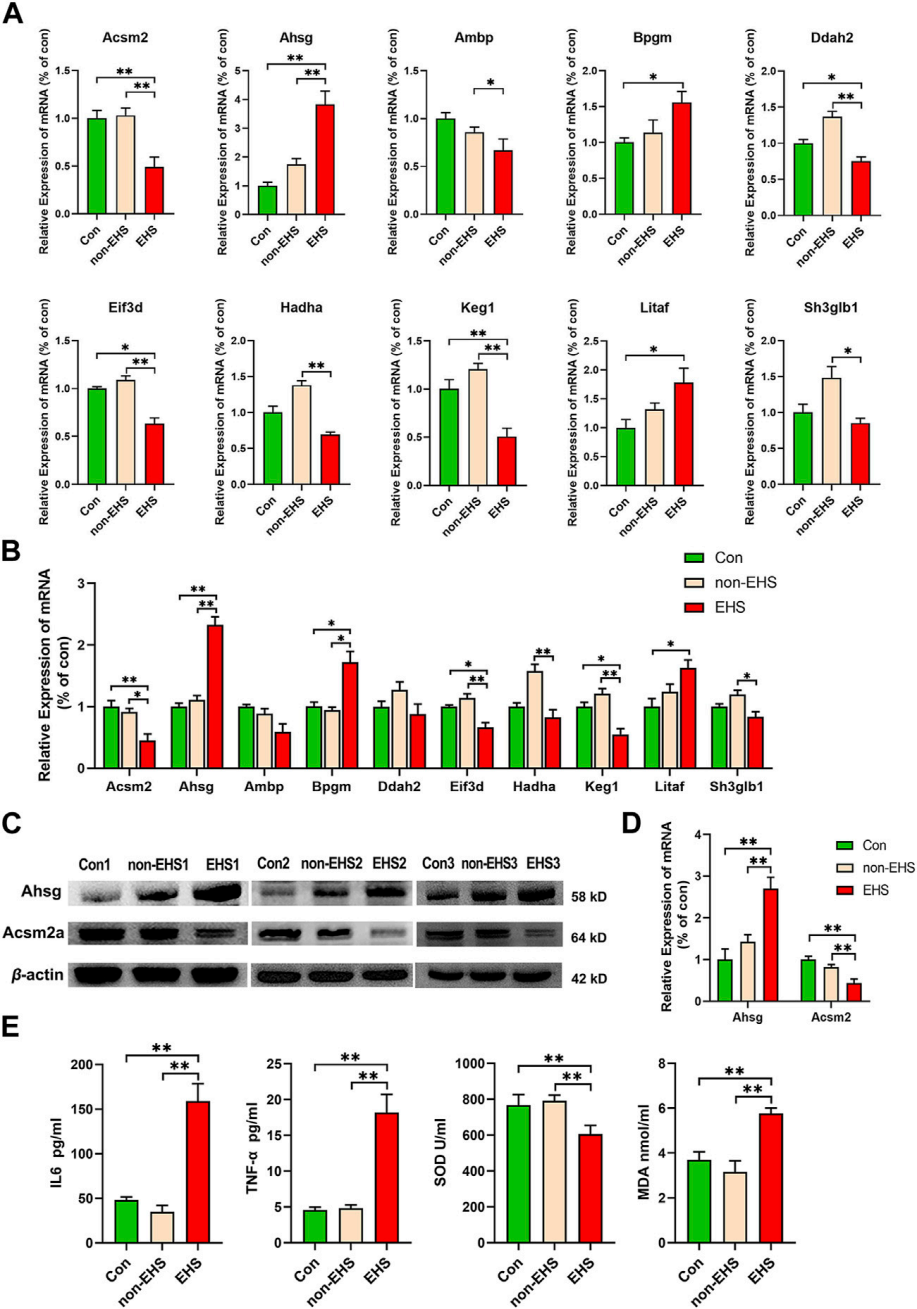
Validation of 10 biomarkers in the kidney and urine of rats with EHS. **(A)** RT-qPCR was applied to the tested mRNAs of 10 biomarkers in the kidney, *p < 0.05, **p < 0.01 (biological replicate = 36, technical replicate = 3). **(B)** RT-qPCR was applied to the tested mRNAs of 10 biomarkers in the urine, *p < 0.05, **p < 0.01 (biological replicate = 24, technical replicate = 3). **(C)** Western blotting was applied to the tested proteins of Acsm2a and Ahsg. **(D)** ImageJ was utilized to analyze the grayscale values of the bands. **(E)** ELISA was used to test the SOD, MDA, IL6 and TNF-α in serum samples, *p < 0.05, **p < 0.01 (biological replicate = 12, technical replicate = 3).

The corresponding primers were designed in accordance with the gene sequences of the 10 significant DEPs that were screened, and qPCR assays were performed on rat kidney and urine. The results verified the significance of their differences, with highly significant differences observed in the expressions of Ahsg, Acsm2, Eif3d, Keg1 (Figures 5A,B). On the basis of qPCR results, one upregulated expression gene Ahsg and one downregulated expression gene Acsm2 were selected for WB validation (Figure 5C). The results show that the high expression of Ahsg and low expression of Acsm2 in the EHS group were consistent with the results of qPCR (Figure 5D). The ELISA analysis of oxidative stress factors (SOD and MDA) and inflammatory cytokines (IL6 and TNF-α) in the retained serum samples demonstrated significant differences between the EHS group and the other two groups. These results further confirm that oxidative stress and inflammatory responses contributed to EHS (Figure 5E).

## 4 Discussion

EHS has become a major cause of sudden death during high-intensity exercise (Arthur et al., 2018; Dubin and Rhee, 2020), and AKI is an EHS-induced multi-organ injury that remains poorly researched. Our primary aim was to identify biomarkers of EHS-induced AKI to provide assistance in monitoring disease progression and predicting possible progression. In this study, proteomics techniques were utilized to screen DEPs in kidney tissues with EHS-induced AKI, and 10 biomarkers were well-screened using bioinformatics analysis and validated by RT-PCR and WB.

The KEGG analysis of DEPs in EHS revealed a confusing result: the high degree of connectivity with the information pathways was associated with Alzheimer’s disease and Parkinson’s disease. This connectivity is corroborated by Bouchama’s viewpoint that neurodegenerative diseases with reduced proteostatic capacity may increase susceptibility to heat stroke, which provides a valid explanation for this connectivity (Bouchama et al., 2022).

Heat stroke induced by high-intensity exercise is closely related to mitochondrial energy metabolism, which was further confirmed by the COG (KOG) analysis of DEPs and subcellular localization carried out in this study (Khan et al., 2018). Fatty acid oxidation (FAO) produces enormous amounts of energy (106 ATP per molecule of FA), whereas carbohydrate oxidation only produces 36 ATP per molecule. FAO is the main source of energy produced by kidney tubular epithelial cells (Simon and Hertig, 2015). Fatty acid metabolism plays an important role in the early changes of kidney injury caused by heat stroke (Xue et al., 2021). AKI induces the abnormal accumulation of lipids in the kidney, and the amount of lipid accumulation is positively correlated with the degree of injury (Xue et al., 2021).

Lipid metabolism in the body is regulated by many factors, and lipid acyl coenzyme A synthase (ACS) is a key enzyme in triglyceride synthesis, including lipogenesis, and a precursor of adipocyte differentiation (Watanabe et al., 2020). ACS is an enzyme that catalyzes the activation of fatty acids and is involved in the first step of fatty acid metabolism, mainly in the outer mitochondrial membrane. Fatty acids are converted into lipids by ACS and then enter the mitochondria to participate in β-oxidation. Currently, the four major classes of lipid coenzyme A are very long chain ACS, long-chain ACS, medium-chain ACS (ACSM), and short-chain ACS. The acyl coenzyme A molecule produced by ACS plays a crucial role in energy production through the activation of FAO in the mitochondria (Khan et al., 2018). ACSM contains five members: ACSM1–ACSM5. The ACSM2A and ACSM2B genes of the human genome are homologous to the mouse Acsm2 (Boomgaarden et al., 2009; Watanabe et al., 2020). The L513S polymorphism in Acsm2 is associated with risk factors (Lindner et al., 2006). In the present study, the significantly downregulated expression of Acsm2 in AKI caused by EHS may be one of the important causes of kidney injury due to abnormal fatty acid metabolism.

The results of serum ELISA show that MDA levels were significantly higher and SOD levels were lower in the EHS group than in other groups, indicating that the rats underwent significant oxidative stress. Mitochondria are the main sites of cellular ATP synthesis, the main organelles for reactive oxygen species (ROS) production, and the center of energy and redox metabolism (Goetzman et al., 2020). Under normal conditions, the lipolysis of TAG into free fatty acids provides energy to the cell. When insufficient lipolysis leads to lipid droplet accumulation, excess lipid droplets in the endoplasmic reticulum lead to oxidative stress and increase ROS levels, causing mitochondrial dysfunction, which in turn promotes the progression of AKI (Lin et al., 2019).

The high number of degrees and log-fold-change values of Ahsg in protein interactions indicates that Ahsg plays an important role in the development of EHS. The qPCR and WB results confirm these findings. Ahsg, as a member of the fetuin family of the cysteine protease inhibitor (cystatin) superfamily (Lebreton et al., 1979) and a plasma glycoprotein synthesized and secreted by hepatocytes, is known as phosphorylated p63 in rats because it is a phosphorylated protein with a molecular weight of 63 kDa (Rauth et al., 1992). It is also known as fetuin A in mice or other mammals (Yamamoto and Sinohara, 1993). Studies have shown that the role of fetuin-A in the etiology of declining renal function through mediating body mass index, uric acid, diabetes mellitus, and hypertension via complex causal pathways (Bassey et al., 2022). Ahsg’s identity as an anti-inflammatory mediator suggested that testing for IL6 and TNF-α inflammatory indicators was necessary. The ELISA results revealed that inflammation did occur and Ahsg might reduce the severity of inflammation by enhancing the phagocytosis of apoptotic cells by macrophages (Klinkhammer et al., 2020; Karadeniz et al., 2022).

Previous studies have shown that fatty acids induce the expression of Ahsg protein, which plays a role in energy homeostasis and adipocyte metabolism (Stefan and Häring, 2013; Gonzalez-Gil and Elizondo-Montemayor, 2020). The Acsm2 protein is likewise one of the important proteins in fatty acid metabolism (Watanabe et al., 2020). This evidence suggested that the expression of Ahsg and Acsm2 was related to the metabolism of fatty acids. The disruption of the intestinal barrier by EHS allows unsaturated fatty acids, which are important components of the cell membrane, to leak into the urinary tract. This phenomenon leads to the elevated levels of these fatty acids in the urinary tract, and thus causes a series of severe reactions. However, identifying the specific mechanism between fatty acid metabolism and AKI caused by EHS should be a key objective of future research.

In this study, the failure to use human clinical samples for validation was a limitation. Low sample sizes occur in many proteomics studies, most likely due to limited funding, and this is a deficiency of this study, although we found interesting differential proteins. In addition, the reason for choosing kidney tissue samples for proteomics analysis was that a low percentage of protein and salt in urine would interfere with proteomic assays, although the testing of urine, plasma, and serum samples may be more relevant for clinical diagnoses. This will be examined further in our future studies. In conclusion, Ahsg, Bpgm, Litaf, Acsm2, Hadha, Keg1, Sh3glb1, Eif3d, Ambp, and Ddah2 were identified as potential biomarkers of AKI caused by EHS, which may help in the future in the clinical management and prognosis of patients, even if our study does not provide direct help for clinical application.

## Data Availability

The original contributions presented in the study are included in the article/Supplementary Materials, further inquiries can be directed to the corresponding authors.
