# Supplementary figures and images for: Proteomics analyses of acute kidney injury biomarkers in a rat exertional heat stroke model
Source: Front Physiol. 2023 Jun 12;14:1176998. doi: 10.3389/fphys.2023.1176998 (PMC10291232; doi:10.3389/fphys.2023.1176998)

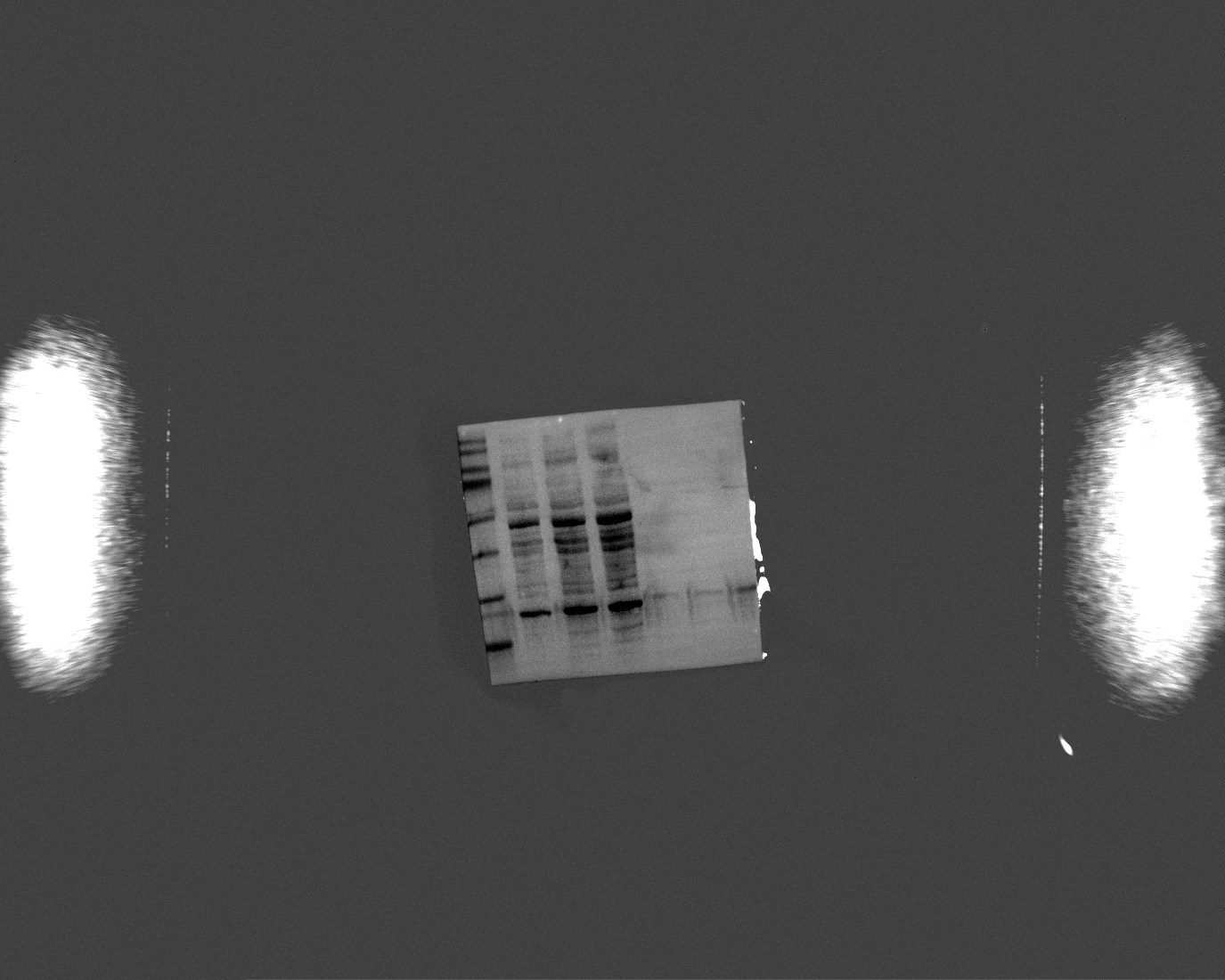

Supplement: Supplementary file 2 [file Image6.TIF]

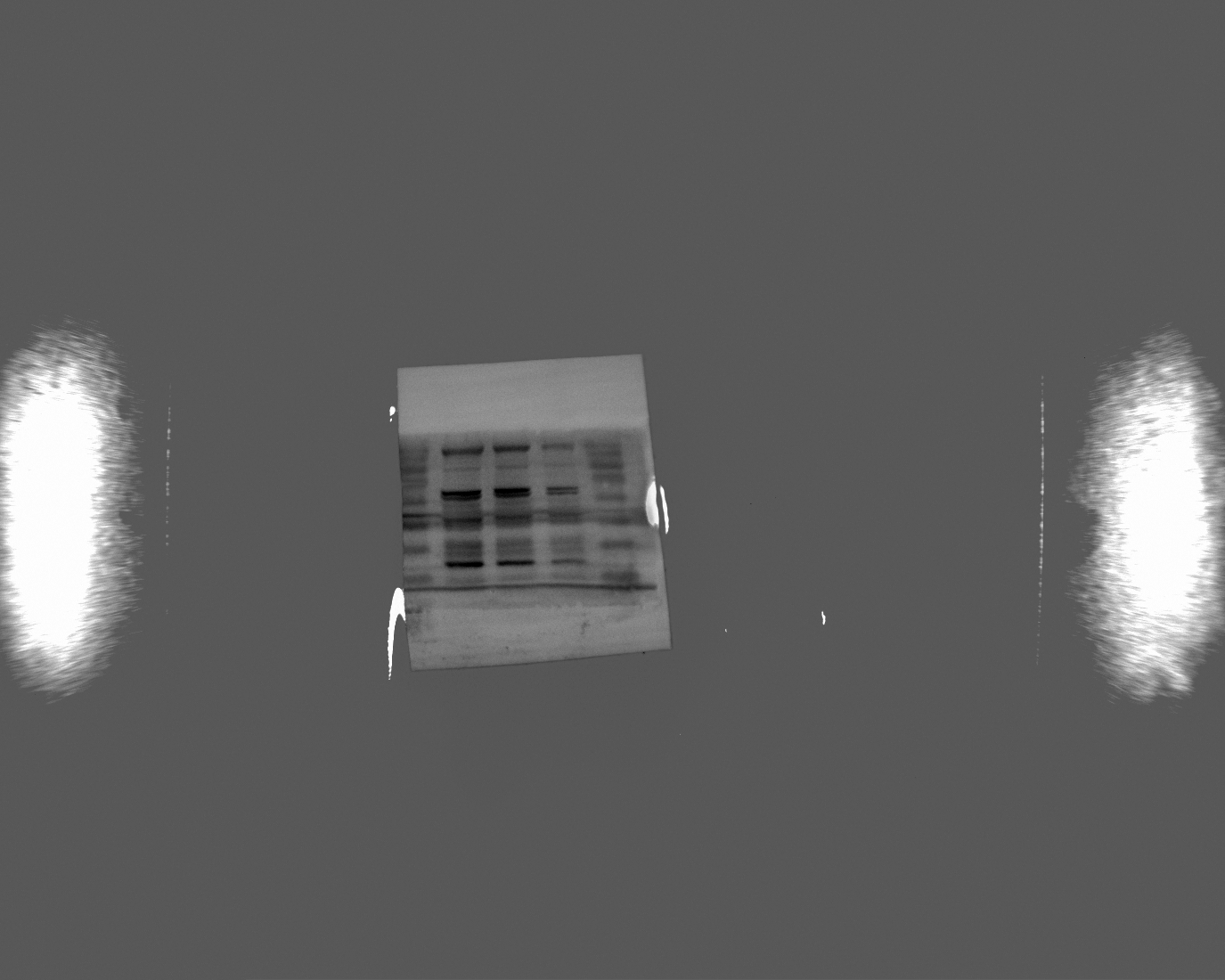

Supplement: Supplementary file 3 [file Image3.TIF]

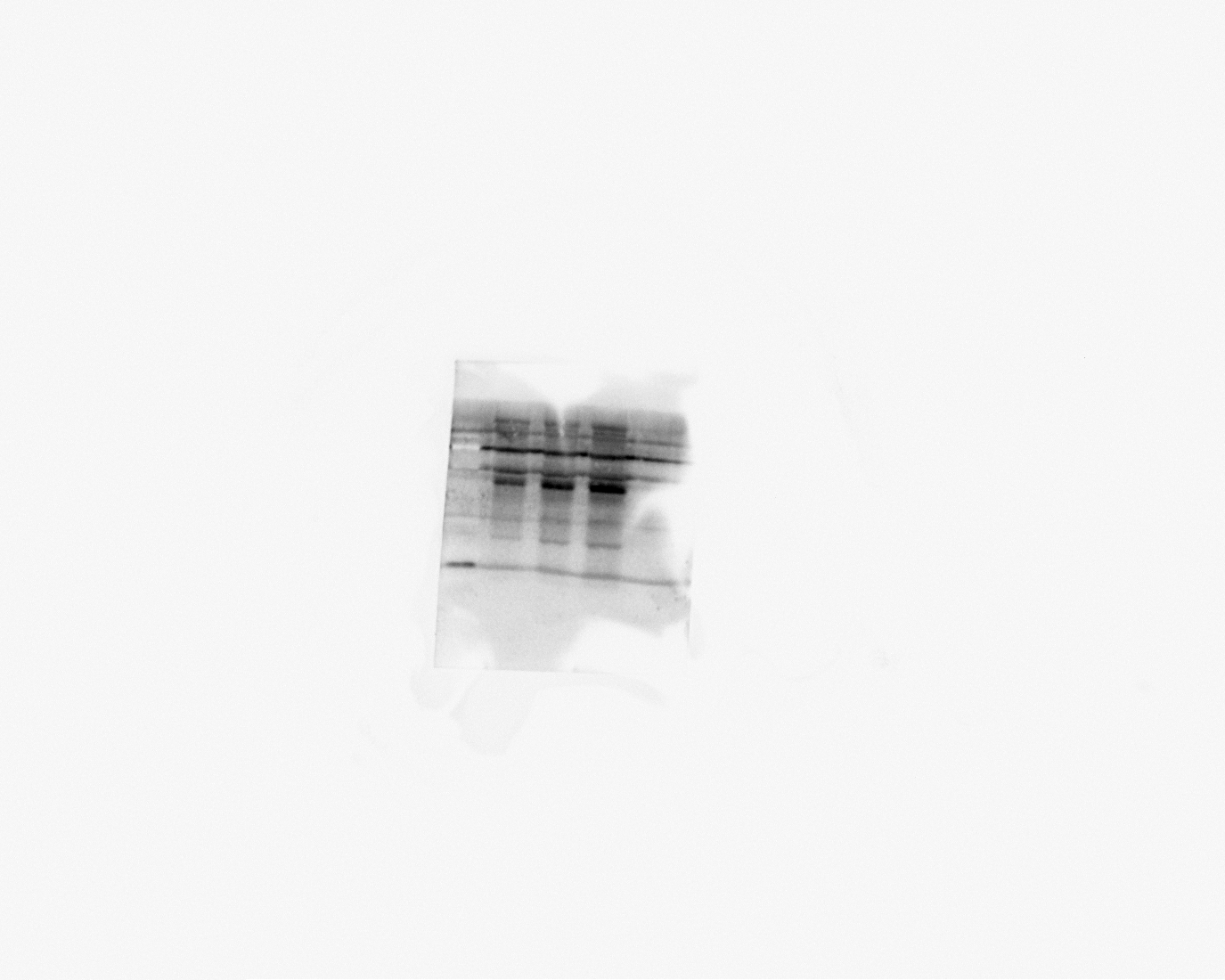

Supplement: Supplementary file 4 [file Image4.TIF]

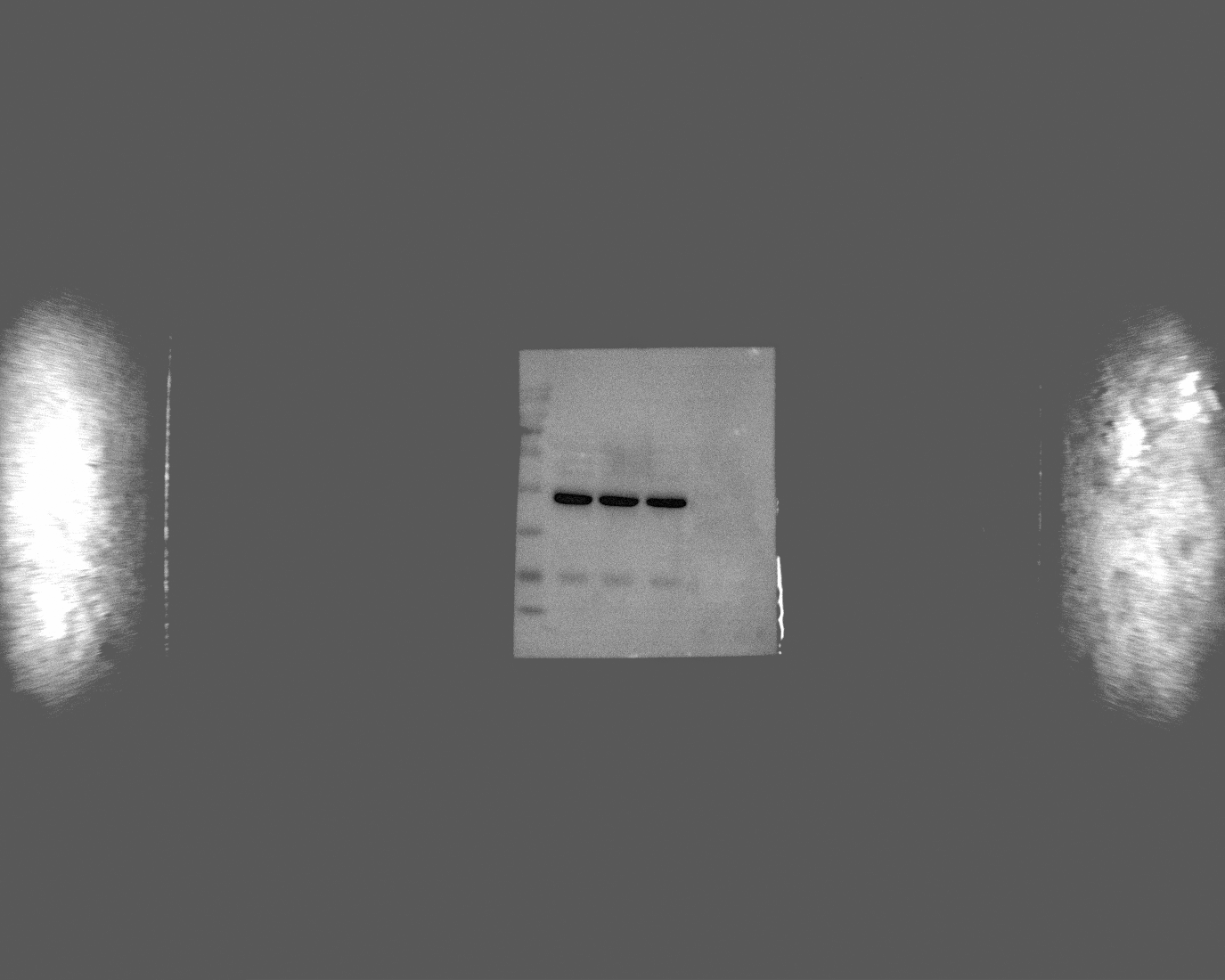

Supplement: Supplementary file 5 [file Image9.TIF]

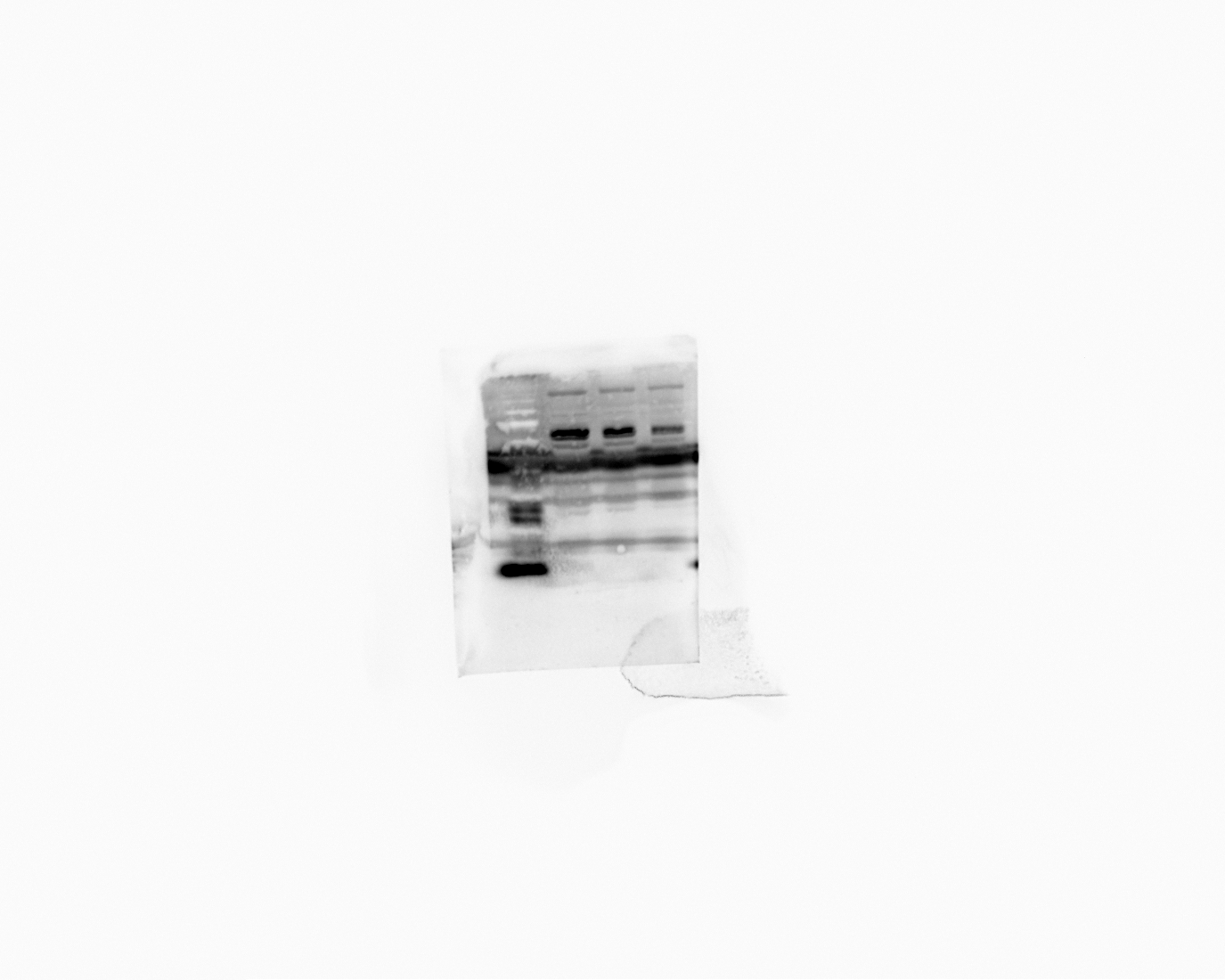

Supplement: Supplementary file 6 [file Image2.TIF]

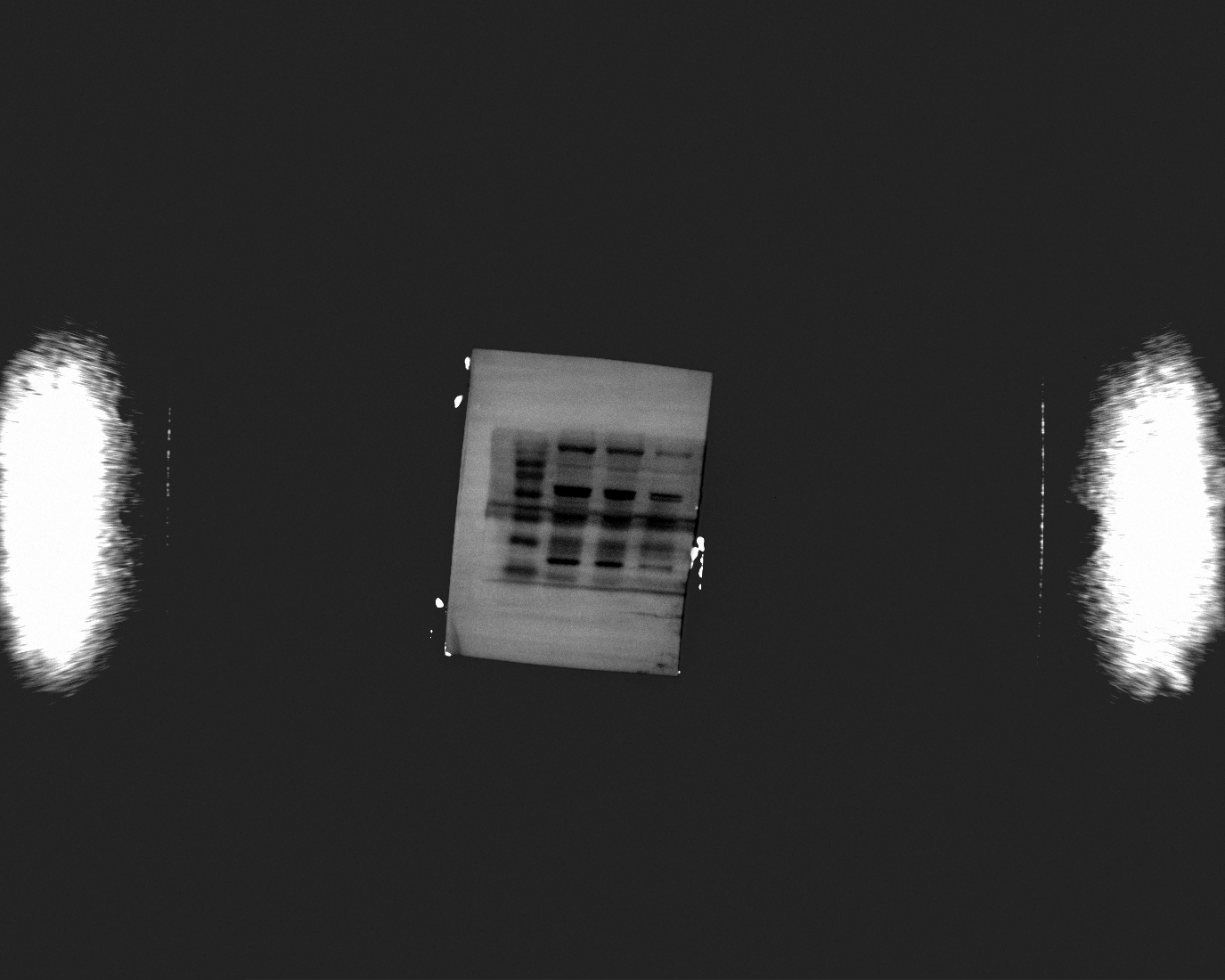

Supplement: Supplementary file 7 [file Image1.TIF]

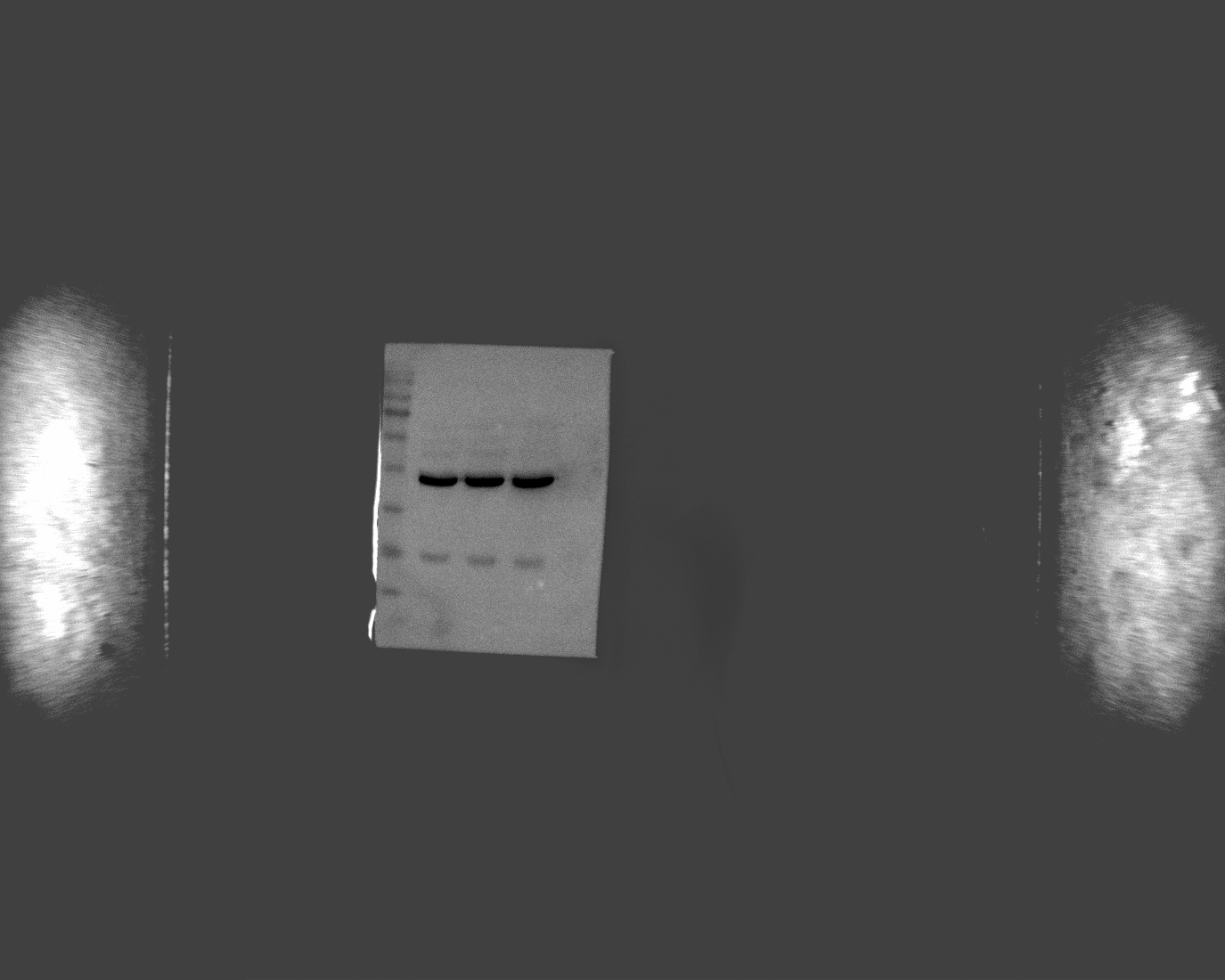

Supplement: Supplementary file 8 [file Image7.TIF]

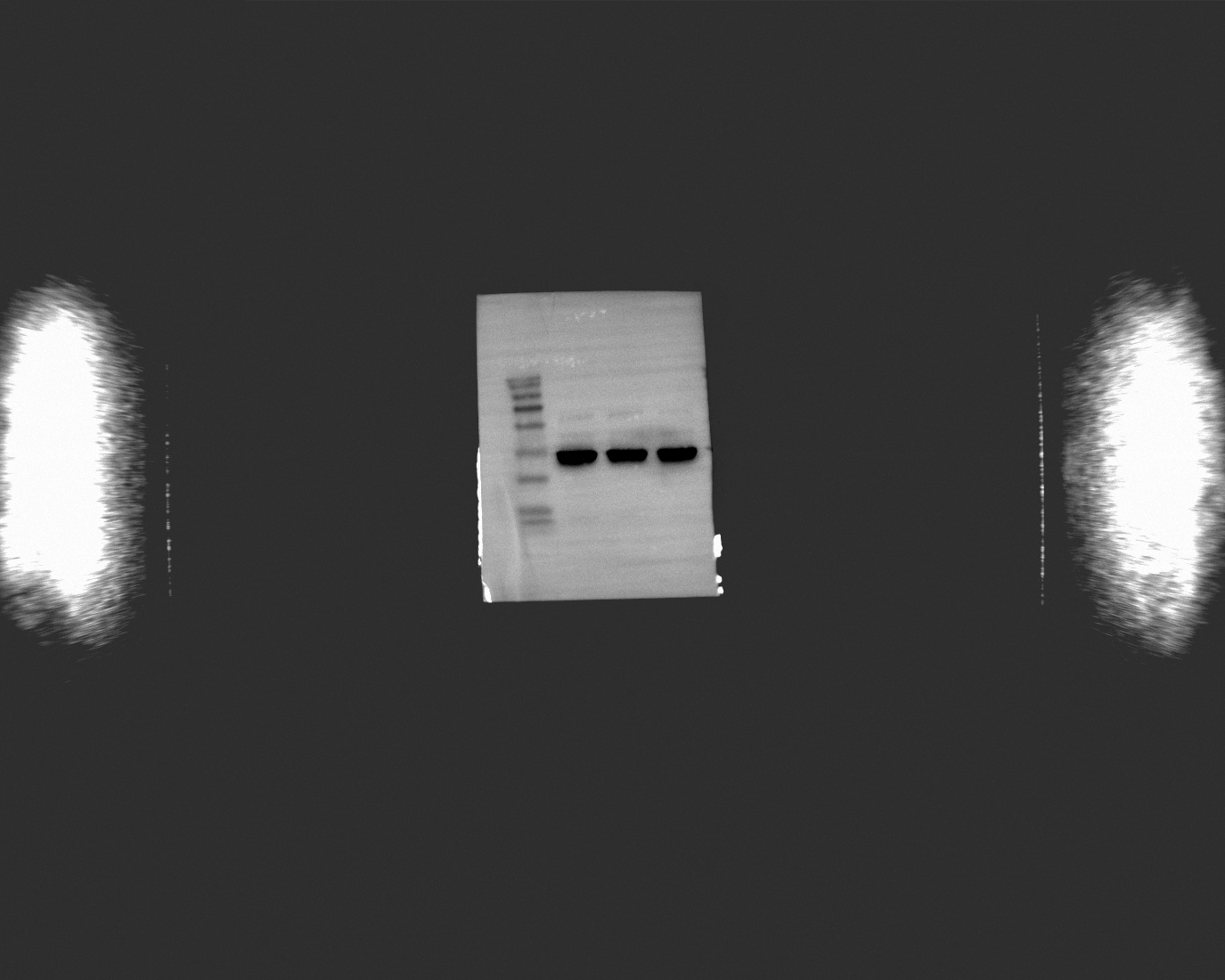

Supplement: Supplementary file 10 [file Image8.TIF]

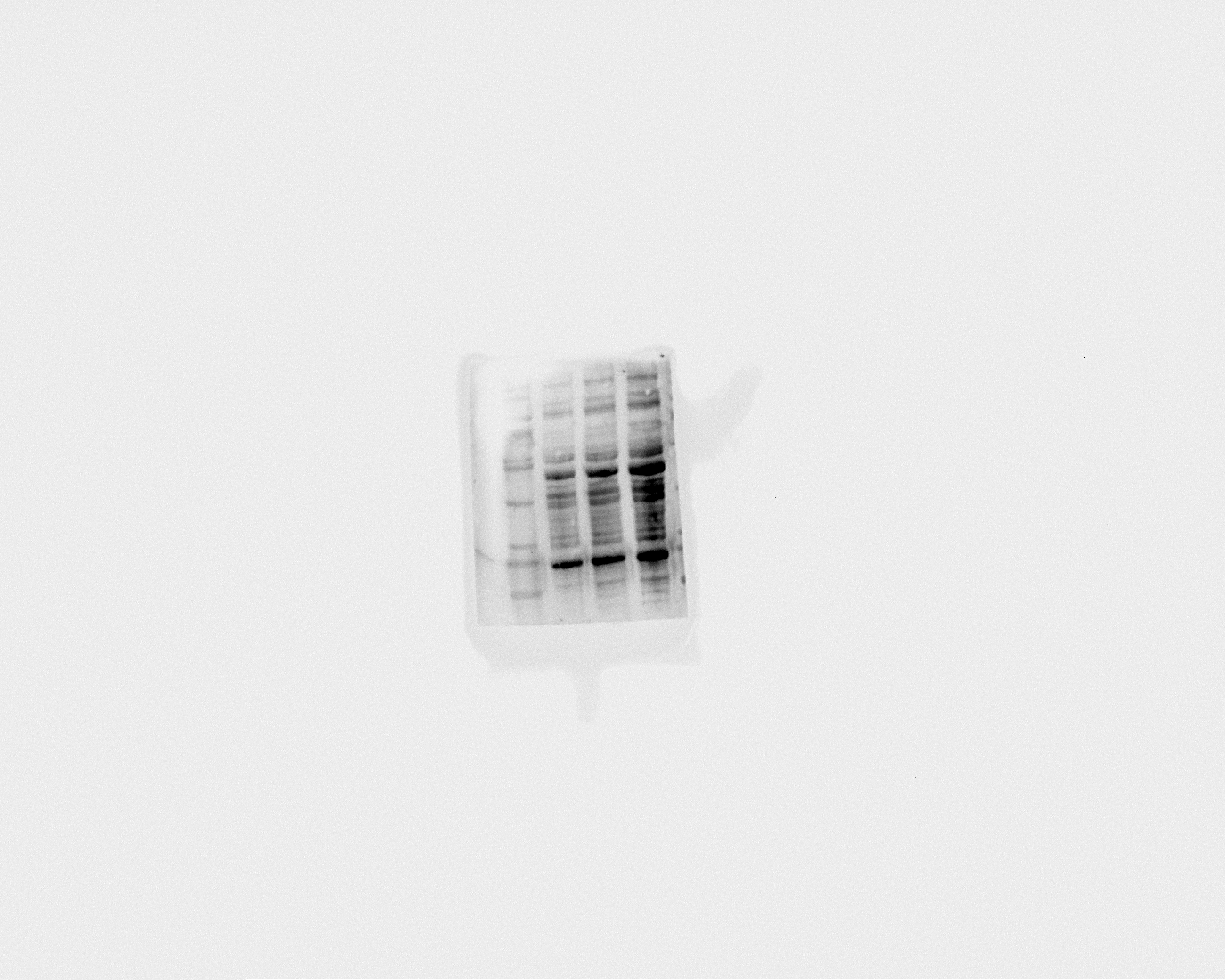

Supplement: Supplementary file 11 [file Image5.TIF]
